# Supplementary material for: Evidence supported by Mendelian randomization: impact on inflammatory factors in knee osteoarthritis
Source: Front Med (Lausanne). 2024 May 28;11:1382836. doi: 10.3389/fmed.2024.1382836 (PMC11165061; doi:10.3389/fmed.2024.1382836)
Supplement: Supplementary file 1 [file Data_Sheet_1.ZIP › data/GCST90274831_5e6/831.forest.pdf]

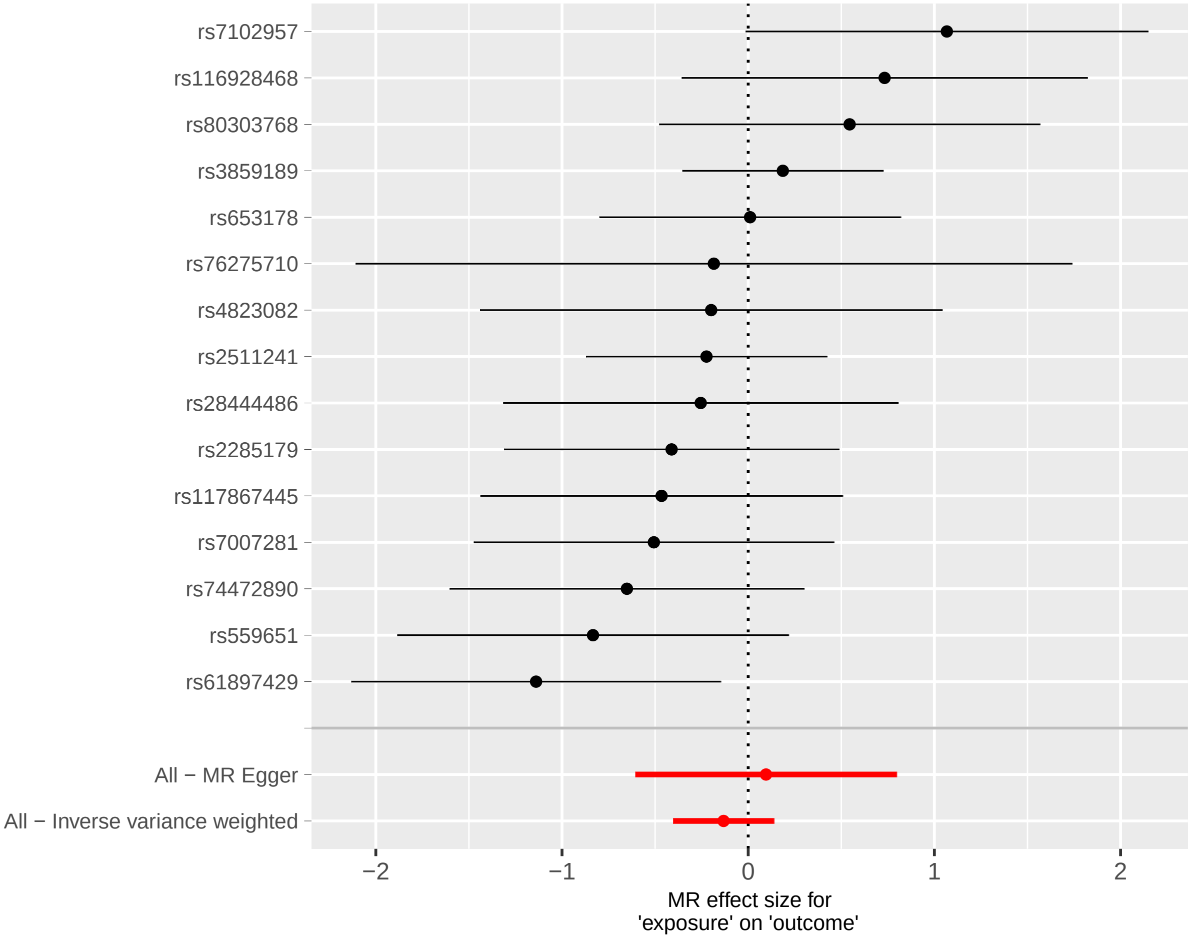
rs7102957
rs116928468
rs80303768
rs3859189
rs653178
rs76275710
rs4823082
rs2511241
rs28444486
rs2285179
rs117867445
rs7007281
rs74472890
rs559651
rs61897429
All − MR Egger
All − Inverse variance weighted
−2
−1
0
1
2
MR effect size for
'exposure' on 'outcome'
